# Design, implementation and performance analysis of an off- grid solar powered system for a Nigerian household

**DOI:** 10.1016/j.mex.2023.102247

**Published:** 2023-06-08

**Authors:** Lambe Mutalub Adesina, Olalekan Ogunbiyi, Kayode Makinde

**Affiliations:** a Department of Electrical and Computer Engineering, Faculty of Engineering and Technology, Kwara State University, Malete, Ilorin, Kwara State, Nigeria; b Department of Electrical Engineering, School of Engineering Technology, Federal Polytechnic Bida, Niger State, Nigeria

**Keywords:** Off-grid pv systems, Performance analysis, Photovoltaic (pv), Power utility, Pv current, Solar power, Solar radiation, Tilt angle, Design, Implementation and Performance Analysis of an Off- grid Solar Powered System for a Nigerian Household.

## Abstract

Solar panel converts direct current obtained from the sun into an alternating current which is often used in various applications. Photovoltaic (PV) power generation technology is used as a stand-alone system to bridge the power demand requirement due to increasing energy consumption. This paper aimed at presenting the design, implementation, and performance analysis of an off- grid solar power system for a Nigerian household. A comprehensive design was done on Solar PV systems, parts and components, and the principle of operation. Average solar irradiance of the location was obtained from data collation center at Nigerian Meteorological Agency (NiMet). The method utilizes the development of block diagram which shows the component layout and their connections and a flowchart which shows the procedure of achieving the objectives of the research. Battery efficiency, PV current measurement, current profile display and commissioning of the installed PV system formed the results. Thereafter, Implementation and performance analysis was carried out. The load demand assessment shows that power required was 23,820 Wh per day at maximum and 11,260 Wh per day when diversity factor was applied (Table 1). Consequently, a 3500VA inverter was selected with a battery size of 800AH.The test result showed that with a load of 11,260 Wh the device supplied energy for about 24hours uninterrupted. Therefore, off grid arrangement reduces the dependency on grid and allows users to derive maximum satisfaction without having relied on public power utilities.

• Obtaining annual solar radiation data from NiMet and determine the load estimation.
• Set up experiment that determine; Battery efficiency, solar panel required and connection mode suitable to achieve the desired current rating, Inverter rating, Charge controller as well appropriate protective devices.
• Installation of project compartments and the commissioning tests by categories of load injection.

## Graphical abstract

**Figure:**
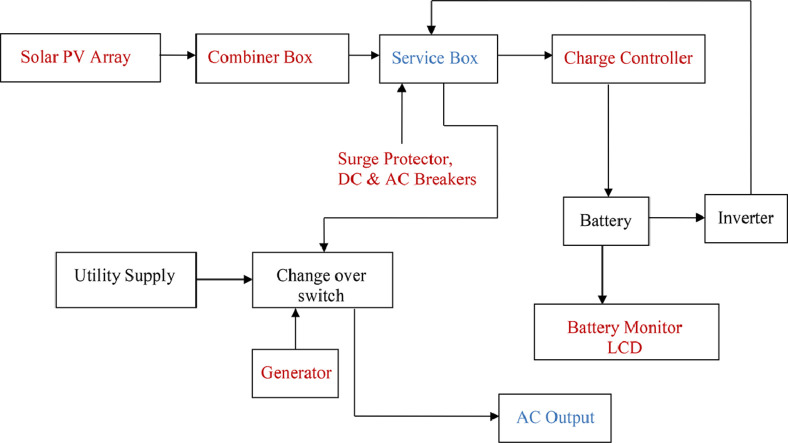

Specifications table

**Table:** 

Subject Area:	Engineering
More specific subject area:	Off-grid solar system
Method name:	Design, Implementation and Performance Analysis of an Off- grid Solar Powered System for a Nigerian Household.
Name and reference of original method:	Not Applicable
Resource availability:	Not Applicable

## Method details

### Background

Nigerian power system is characterized with continuous power supply failure and fluctuations. The fluctuation affects sensitive equipment while the continuous failure or blackout creates sadness among the generality of the citizen. The alternative source of electric power supply that alleviate these scenarios described above is renewable energy (RE) [1]. Solar energy (SE) is a typical example that are very common, they are clean and readily available in abundant. SE is actually the extraction of energy from the sunlight and subsequently converts it to usable energy. The RE in developing areas is often found more affordable in cost implication than the energy obtained from the convectional electricity obtained from power utility [1,2].

Solar power generation components comprise of solar panel, inverter, charge controller, rechargeable batteries, cables and protective devices like surge protector [3]. The inverters are generally classified into two; the self - excited oscillation inverters and external excited oscillation inverters. The varying intensity of the sun during the day may create an over-charging particularly where the panels are connected directly to the battery hence the need for a charge controller [4]. The benefits of solar power include; huge savings on power utility bill via optimization of power inverter, solar hybrid inverter enables charging of batteries through the PV panel which also reduce further the power utility bills, cost of running petrol generator is drastically reduced, low maintenance cost compared to using ac generator or conventional power supply, reservation of power for night when there is no sunlight via batteries storage [5].

This research is aimed at carrying out design and performance analysis of an Off - grid solar powered system. The specific objective (s) is to develop a standard procedure for the design and performance analysis of an Off - grid solar powered system, subject the developed procedure to test for a case study of 3.5 kVA Off - grid solar PV system in Ilorin Kwara State, to tabulate PV Voltage, PV Current, and Battery Voltage with respect to time for a period of 6 months and obtain it average and to carry out performance analysis on for off grid solar PV system using suitable solar cell curve characteristics.

Ogbekwe (2010) designed a 3- kVA hybrid power supply system to assist in Computer Laboratory Experiment [6]. A 560 W, 24 V solar panel as well as PWM charge controller specifications were used for making hybrid system. When this was compared to other source such as electricity supply, it was discovered that the approach increased the reliability of the power to the consumer. The configuration made does not match the estimated load and thus, the batteries were not sufficiently charged on daily basis. In the same development, the PWM controller selected couldn't convert the over-voltages to equivalent current. Allouhi, et al. (2016) carried out the design of a grid - connected PV systems which is installed on school building [7]. The research draws a technology comparison, energy analysis and economic performance for the two types of solar panels in consideration. Comparison was made between poly and mono solar panels, and it was discovered that poly has more monthly yield than mono. The reliability of the system was not 100% because either of the two can fail which imply that there was no provision for back-up in case of eventuality. The proposed research work would feature the off-grid solar PV system, utility power supply and Petrol driven generator so that if anyone fails other alternatives can serve.

Ghenaia and Bettayebb (2019) presented a hybrid solar PV system design that comprises of a grid - tied solar PV and fuel cell for university building usage [8]. It is aimed at designing a grid connected RE system such that the building load is met with high penetration of RE that resulted into low greenhouse gas emissions and low cost of energy. It was discovered that the percentage of fuel cell was to the tune of 32% which is still harmful to the immediate environment due to the gas emission produced from the fuel cells. This is different from the new design because percentage of gas emission is less to 5%. Gerard (2020) carried out the researched-on application of RE as alternative option to the conventional utility electricity supply to a faculty building in one of the Nigerian Universities [9]. Microsoft excel and PVsyst V6.55 were used to carry out comparative analysis. The analyzed results of 400 kW solar panels/batteries, conventional power utility supply of the area and a 500kVA generator set shows that, for duration of 25 years, the generator set system is cost effective over the conventional power utility supply and solar system. This analysis further displayed that large amount of gas emission from the generator will definitely pollute the environment and cause hazard to the immediate neighbors.

Chaudhry and Iqbal (2021) designed an off - grid solar PV plant for small scale application. Comparing the performance with a conventional electricity supply [10], it was observed that an off - grid PV system operation is more costly after a long-time usage consideration. However, there was no provision for service box for maintenance and repairs in case the need arises. Michael (2012) carried out an off - grid solar system designed and analysis for a DC Load in Pakistan [11]. The researcher established that the off-grid solar PV electricity is less costly than the conventional power utility supply considering the prevailing carbon tax and price of oil. But the design excludes AC loads meaning that the design was purely carried out for DC loads only.

## Material and method

Based on the method of electric energy production or generation, PV modules are arranged in arrays to get the desired electric output. Solar PV systems are classified based on their functional and operational requirements as well as their components configurations [12]. PV systems are therefore classified into off - grid solar PV Systems (stand -alone solar PV), grid - connected PV Systems and Grid - Connected with Power Backup [12,13].

The standard of living is improving on daily basis which necessitate the demands for large amount of energy from conventional source. Consumers show more interest in off-grid network arrangement relatively all over the world. By this action, the system is decentralized and thus requires less space or land than a conventional energy generation, no or less distance - related transmission losses and gives the same electricity as a conventional connection. Off-grid systems also supports the integration of decentralized renewable generation into the grid and thus ensure power stability and reliability which of course reduced power outages and impact on economic activities [13].

**Fig. 1:**
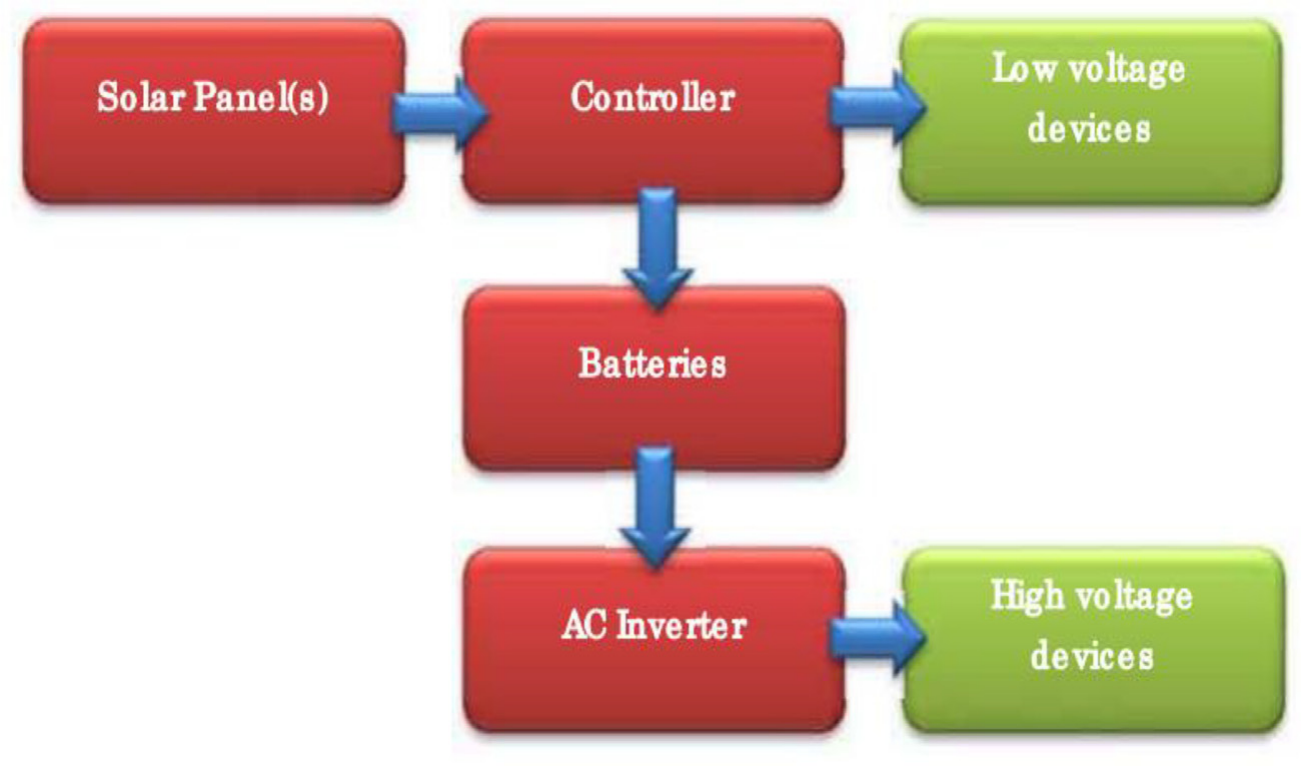
Off - Grid PV System [14].

**Off- Grid Solar PV Systems:** An off - grid solar PV Systems (or a stand - alone solar PV systems) are designed and sized to supply dc and/or ac electrical loads. Some stand - alone PV systems are sometimes called direct coupled PV system if the dc output of the PV module array is directly connected to a dc load. In Such connections, there is no electrical storage (batteries), hence, the load operates only during sunlight hours. However, the other type of off - grid solar PV systems have components like maximum power point tracker (MPPT) called charge controller connected between the array and load which assist in array maximum power output and in matching the impedance of the electrical load to the maximum power output of the PV array. The MPPT and PV panels are connected in series to have the desired increase in dc voltage, such. 12 V, 24 V or 48 V. Other connected components include batteries and inverter. This set-up of off-grid PV systems is not connected to distribution network of power utility company as shown in Fig. 1.

The Fig. 1 provides both low-voltage DC power for running smaller electrical devices and appliances such as laptop computers and lighting, plus a higher-voltage AC supply for running larger devices such as larger televisions and kitchen appliances. In this diagram, the arrows show the flow of current. The solar panels provide the energy, which is fed into the solar controller. The solar controller charges the batteries. The figure also illustrates how a controller supplies power to devices using either the solar panels or the batteries as the source of this power. The battery stores the excess electricity generated by the solar panels. The AC inverter takes its power directly from the battery and supplies the high-voltage devices.

**Fig. 2:**
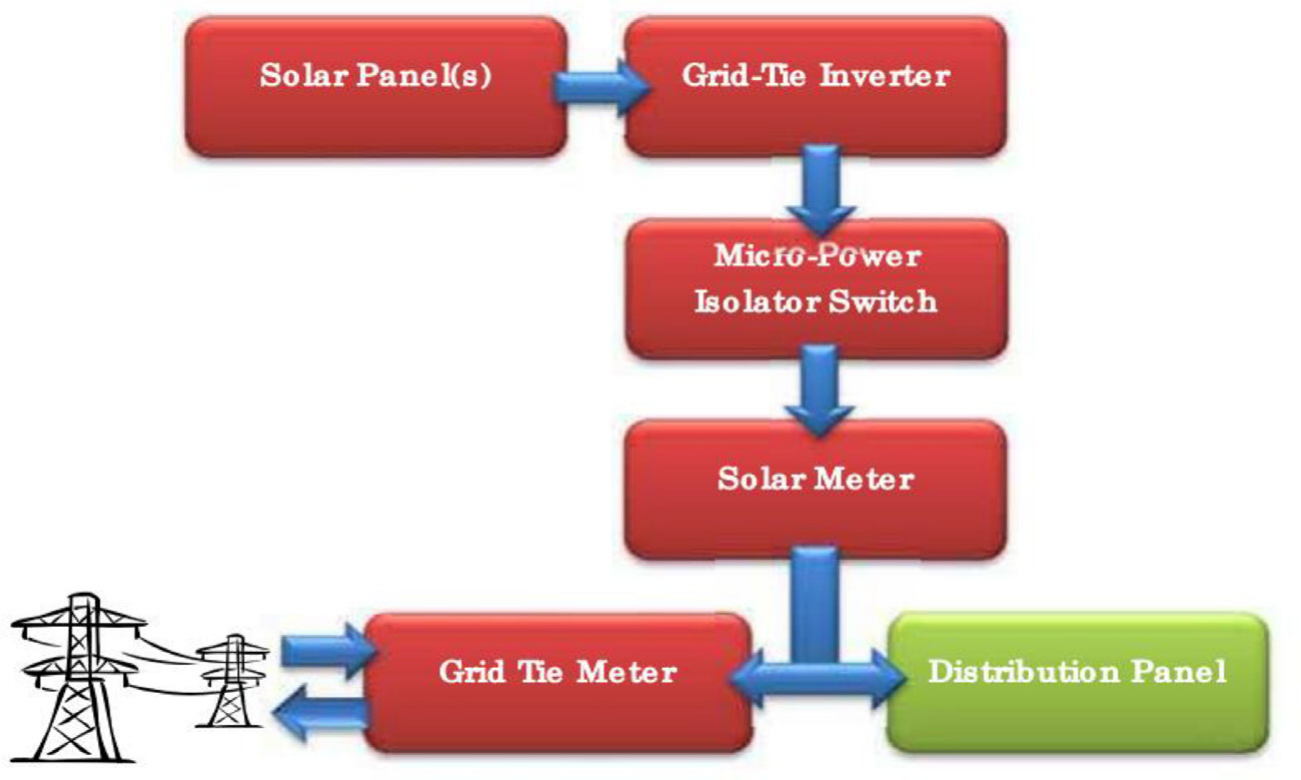
Grid - Connected PV System [14].

**Grid - Connected PV Systems:** In a grid - connected PV systems, the power conditioning unit (PCU) called grid - tie inverter converts the dc power produced by the PV array into ac power considering the voltage and other quality requirements of the power utility network in question. A two -directional interface is required between the PV system ac output arrangement or circuit and the power utility network. This allows the ac power produced by the PV system to either supply the designed loads or be used to back feed the grid when the PV system output is greater than the designed load demand. In the same way, the PV system also receives back-up power from power utility grid when the PV system is not producing enough power. Consequently, service meters are required to record power consumption at the service point [15]. Inverters remain the main difference between a grid - connected system and a stand - alone system. In this case, Inverters must have line frequency synchronization capability to deliver the excess power to the grid. If a net material used, they must have capability to record the power consumed or generated power in an exclusive summation format. Fig. 2 illustrate a grid - connected PV Systems.

In this figure, the solar panels are connected to the grid-tie inverter, which feeds the energy into the micro-power isolator switch. The micro-power isolator switch is seeing as one of the safest means of manual isolation between grid-tie inverter and solar meter, and it is suitable for all grid-tied and off-grid installations. Electricity can be used by the devices in the building or fed onto the grid depending on demand. The grid-tie inverter monitors the power feed from the grid. If it detects a power cut, it also cuts power from the solar panels to ensure that no energy is fed back into the grid. The solar meter records how much energy is generated by the solar panels while the ‘grid tie meter’ monitors how much energy is taken from the grid or fed back into the grid using the solar energy system. The distribution panel connects the system to the electrical grid. The basic function of arrows in this diagram is to show direction of flow of current.

**Grid - Connected PV Systems with Power Backup:** Grid - connected with power backup which is called grid interactive system. It forms a combination of a grid - connected PV systems installation and a bank of batteries. Recall that in grid - connected PV Systems, the loads use power from the solar array when the sun shines and the excess are sold to the power utility customers on the grid. In this case, the battery bank provides contingency for the power cuts so that one can continue to use power from the system.

### PV system

The PV system is made up of components that comprises of inverters, array of panels, charge controller, battery, and protective devices. All these components are inter-connected form a PV system. Details of these components are fully described in the next subsection.

### Description of photovoltaic components

**AC Loads**: These are electrical appliances or connectable equipment used in households such as lights, fridge, fans, cookers etc. Determination of a household load is an important factor in design and implementation of a stand - alone solar PV system. In household load determination, Eq. (1) is often used to calculate the average daily load consumption [2,4].

(1) $$A1=\;\Sigma_{i=1}^{n}\frac{Li\ldots n\times N\times D\times H}{K\times \;7}\;$$

Where;

 A1= Average energy demand per day

 L_i…n_= Individual load Wattage

 N= Number of individual loads

 H= Number of usage hours per day

 D= Number of usage days per week

 K= inverter efficiency.

This power adjustment factor (K) otherwise known as inverter efficiency varies between 0.8 and 1 depending on the maker of the inverters. Also, inverter efficiency depends on the preliminary test results of the Battery efficiency experiment which is necessary where batteries are purchased and kept for later use. In practice, k is taken to be 1. The essence of dividing Eq. (1) by 7 is to obtain an average weekly consumption of each load.

**Inverter:** Inverters are simply described as AC Drives, or variable frequency drive (VFD). It is capable of turning Direct Current (DC) to Alternating Current (AC). Its controls the speed and torque for electric motors and are rated in Kilo volt-amperes (KVA) or volt-amperes (VA). Inverters are usually designed about 25% higher than the calculated connected loads [2,5]. Eq. (2) and (3) illustrates the **connected** or total household wattage (B1) and the inverter rating specification formula (B2) [2,4]. The inverter outputs are classified in to three; namely, sine, modified sine, and square waves. In practice, sine wave can be obtained in both power utility company and generator.

(2) $$B1=\;\frac{A1}{\text{Total}\;\text{hours}\;\text{of}\;\text{usage}}$$

(3) $$B2=\frac{\text{Total}\;\text{wattage}}{\text{Power}\;\text{factor}}$$

Where;

Powerfactor(PF)=0.85

Therefore,

(4) SelectedInverter,(B3)=B2+(30%ofB2)

For cost implication,

(5) CostofInverterRequired,(B4)=No.ofSelectedinverter(B3)×CostperInverter

**Batteries**: Batteries are source of electric power with each of them consist of one or more electrochemical cells. A battery has external connections for powering electrical circuit or devices. The positive terminal and negative terminal are called anode and cathode respectively. Batteries become necessary in solar design and implementation because the sun does not always shine and the acquired energy from the sun needs to be kept or stored somewhere for various applications. The batteries are obtainable in different categories depending on their ratings and manufacturer as well as classification based on the areas of its applications. While in operation, PV batteries normally undergo charging and discharging processes. Available Battery types often used in Solar PV applications include; Lead - acid, gel-type lead - acid and Nickel-Cadmium or Ni-Metal hydroxide batteries. Among all, lead-acid batteries are commonly used because of their deep discharge. A battery life span is from 3 to 5 years [1], [2], [3], [4], [5], [6] and rely on variable like temperature, charging cycles and discharging cycles [2], [3], [4], [5], [6], [7], [8], [9], [10], [11]. PV battery parameters for solar power design and implementation are calculated using Eqs. (6) to (13) [2].

(6) $$Average\;amp-hour\;demand\;per\;day\;\left(C1\right)=\;\frac{Average\;energy\;demand\;per\;day\;\left(A1\right)}{inverter\;voltage}$$

(7) $$Required\;battery\;capacity\;\left(C2\right)=\;\frac{C1\;\times \;D.O.A}{D.O.D\;\times \;0.85}$$

Account for system losses = 0.85

(8) $$No.\;of\;batteries\;in\;parallel\;\left(C3\right)=\;\frac{C2}{Amp-Hour\;capacity\;of\;selected\;battery}$$

(9) $$No.\;of\;batteries\;in\;series\;\left(C4\right)=\;\frac{Inverter\;voltage}{selected\;battery\;voltage}$$

(10) Totalnumberofbatteries(C5)=C3×C4

(11) Totalbatteryamp−hourcapacity(C6)=G

(12) G=C3×Amp−hourcapacityofselectedbattery

(13) $$Total\;battery\;kilowatts\;hour\;capacity\;\left(C7\right)=\;\frac{C6\;\times \;Norminal\;Voltage}{1000}$$

(14) CostofBattery(C8)=No.ofBatteriesRequired×CostperBattery

**PV Array:** Solar panel received sunlight energy and convert to useful dc electricity. They are classified based on manufacturer makes and power generation. Solar panels are made of crystalline silicon which are either mono-crystalline or poly-crystalline silicon. Mono-crystalline panels are cut at four sides to make cylindrical silicon wafers. Modern panels are made of thin film which are regarded as emerging photovoltaic because of its developmental stages [13]. Therefore, a solar PV array involves multiple of panels that are interconnected to have a desired power generation. This is because the quantity of solar power generated by a single PV panel may not be enough to suit the proposed applications.

Standard PV panels having output voltage of 12 V, 24 V, or 48 V are often produced by the manufacturers. For a higher voltage requirement, more than one panels are series connected. While for a higher current requirement, more than one panels are parallel connected producing the needed power output. Therefore, in PV systems, panels can be connected in series, parallel, and series - parallel. To have a satisfactory overall system efficiency, experience shows that panels of different manufacturers are advised not to be mixed together as single array connection even when their parameter specification are the same. The reasons are not unconnected with the possible mismatch losses due to differences in solar cell characteristic curves and their spectral response.

Therefore, in determining the required solar panels or array, the solar irradiance and sunshine-hour of the choosing case study are used. Eqs. (15) to (22) are used in this process:

(15) PhotovoltaicmoduleCapacity(D1)=(B1X1.3)/(PGF+PSH)

Where;

 PSH= Peak sun hour.

 PGF= Panel generating factor.

 1.3= Specified energy lost in the system

Whereas;

(16) PGF=SIxTCF

SI=Solar irradiance

TCF is the total correction factor of the panels.

Therefore, for solar panels, TCF = 0.62 [2]

(17) $$No.\;of\;Modules\;needed\;to\;meet\;requirement\;\left(D2\right)=\;\frac{Required\;PVmodule\;capacity}{Selected\;PVmodule\;wattage}$$

(18) $$No.\;of\;Modules\;req.\;per\;string\;\left(D3\right)=\;\frac{Nominal\;battery\;voltage}{selected\;PV\;module\;power\;voltage\;at\;STC}$$

(19) $$No.\;of\;strings\;in\;parallel\;\left(E1\right)=\;\frac{C2}{Number\;of\;Modules\;per\;string}$$

(20) TotalNo.ofModulesneeded(E2)=Z1xZ2

Where;

Z1= Number of modules per string

Z2= Number of parallel strings.

(21) ArrayOutputRating(E3)=PVmoduleOutputRating×Panelsrequired

(22) TotalCostofModules(E4)=Numberofmodulesrequired×CostPerModule

Eq. (17) and (20) provides the same answers. This implies that any of the two approaches can be applied to evaluate the modules needed to meet the required loads.

**Charge Controller (CC)**: This is a battery regulator which limits the rate at which electric current is added to or drawn from the batteries. Charge controller prevents both overcharging and voltage of batteries which can reduce the battery performance and lifespan or risk of losing the entire system due to excessive heat. It also prevents battery from deep discharging.

In solar PV panels, charge controllers regulate output power or the dc voltage output of the batteries. This output voltage serves as input voltage to the charge controller and thus converts such to the same dc voltage needed for battery charging. Charge Controller of MPPT scheme are often used because they maximize output efficiency. MPPT is a micro-controllers-based equipment which compute an out power at any given time with voltage under monitoring and regulation and the out power remain stable. Thus, a charge controller enhanced the power transfer efficiency.

As an illustration, consider as example of a 48 V rating solar charge controllers having a maximum voltage (Voc) of 150 V. Therefore, based on earlier discussion in Array panel above, a maximum of 3 panels will be allowed to be connected in series. Consequently, in selecting an appropriate charge controller, Eq. (23) and (24) simplifies the task.

(23) Chargecontrollercurrentrating(M1)=Isc×E1×1.25

(24) Chargecontrollervoltagerating(M2)=Voc×D3

Where;

Isc= PV module short circuit current

Voc= PV module open circuit voltage

**PV Cables:** Solar cable specification varies from one country to another because of the change in terrain and weather. Notwithstanding, required cables for installation are in the following classes of applications; namely, overhead lines, power, and control cables as well as the general accessories.

Cable connections in solar PV are made between the solar panel arrays and between the panel and other solar PV components. These cables are made in British as well as other International Standards. For example, EN50618 standard cable which very often are typically use for rooftop solar installations and solar farms makes interconnection of solar PV system's components easy. This cable is an outdoor cable and made to withstand varying environmental conditions and all forms of degradation arising from its exposure to Ultraviolet light. Furthermore, bar copper conductors are also used for earthing of the necessary solar PV components. The EN50618 solar cable has TUV certification having undergone a serious process of manufacturing and testing to ensure quality cable with lifespan of twenty-five years is guaranteed.

Also, the article 690.31 of national electric code (NEC) stipulate that PV systems should be connected using single conductor cable type or the available single conductor cable which is labeled as PV wire [15,16]. Although, aluminum conductors and copper are sometimes used in PV system installation and are made as a stranded or solid conductor. Stranded cables are for larger size of solar PV system installation because of their better conductivity than solid cables and their extended cable surface [11,16].

**Solar Mounting Structure:** This is a location at the solar PV site that accommodates arrays of panel so that sun reaches its surface at a selected angle. Typical of these mounting structures are Pole top, Ground, Pole side, Roof of Building, and Tracking. Each of the mounting style have their disadvantages ranging from long length of PV cable requirement and so on. For example, in roof mounting type, panels are placed on the roof of the building. Cables are often laid between the battery bank and arrays of panel, and such length of cable are in most cases small in quantity. Whereas in some other application the length of cable is more in quantity. Track mounting structures for solar arrays are quite preferable because of their high efficiency as a result of having the opportunity to use the whole day sunlight [[17], [18], [19]].

**Combiner Box:** Where large number of solar panels is used, their wiring connections required that one or more boxes be placed near the panels which will combines several of these interconnected wires from solar panels to a few wires for the connection to the charge controller.

**Service Box:** The service box comprises of surge protector with a prescription ‘XLSDP’ to divert any surge occurrence. The alternating current (AC) and direct current (DC) breakers are used for overload protections, electrical short circuit as well as needed for repairs and proposed future maintenance.

**Surge Protectors:** It protect the PV system from power surges that often come up when the system or any part of the network is struck by lightning. A power surge is a sudden s increase in supply voltage far above the rated voltage.

**Battery Monitor:** Some battery monitors are with a built-in liquid crystal display (LCD) unit. These are often used to monitor the level of individual battery.

**Generator:** This is an alternative source of electric power supply whenever the sun is either not available at all for the day or not available for long, and perhaps in addition the batteries are down totally. With any of these mentioned conditions, generator will provide electricity for the purpose.

**Change Over Switch (COS):** This is a transfer switch between all the three sources of power supply; namely, the power utility source, generator, and solar power. Thus, COS is used to transfer power supply from available power source to the load.

**Utility Power Supply:** This is an electricity supply from national grid which power source may be from any of these generation stations; hydro, thermal, or gas station or combination of all.

**Grounding Process**: The grounding system facilitate low resistance between the solar PV system and ground [20]. Therefore, the solar system is shielded from current surges emanating from circuit switching, system break down and lightning strikes. The grounding also serves as a reference point and ensures system balanced voltages.

### Methodology of design and implementation of 3.5KVA solar pv system

**Fig. 3:**
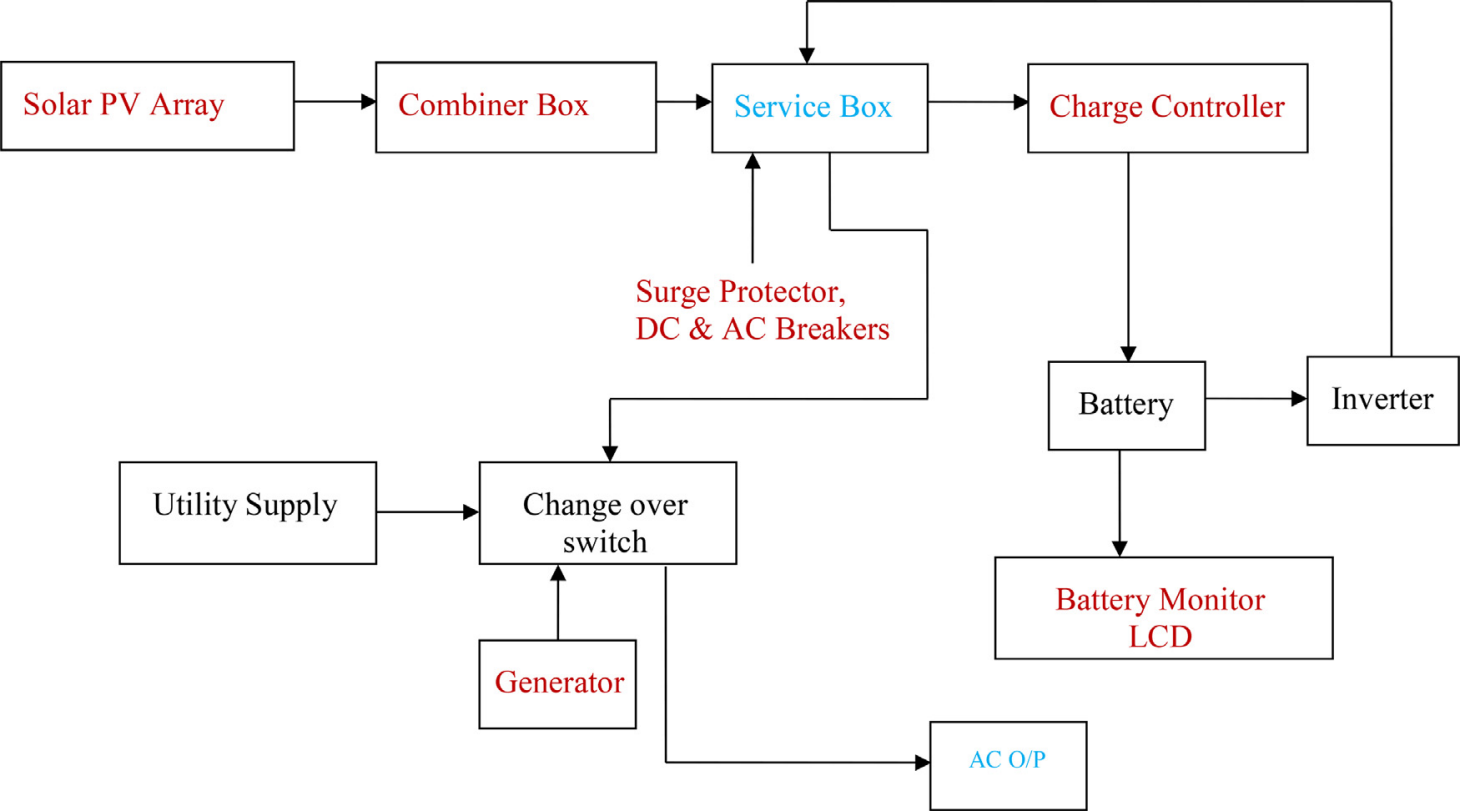
Block Diagram Representing Components of Off - Grid System.

**Fig. 4:**
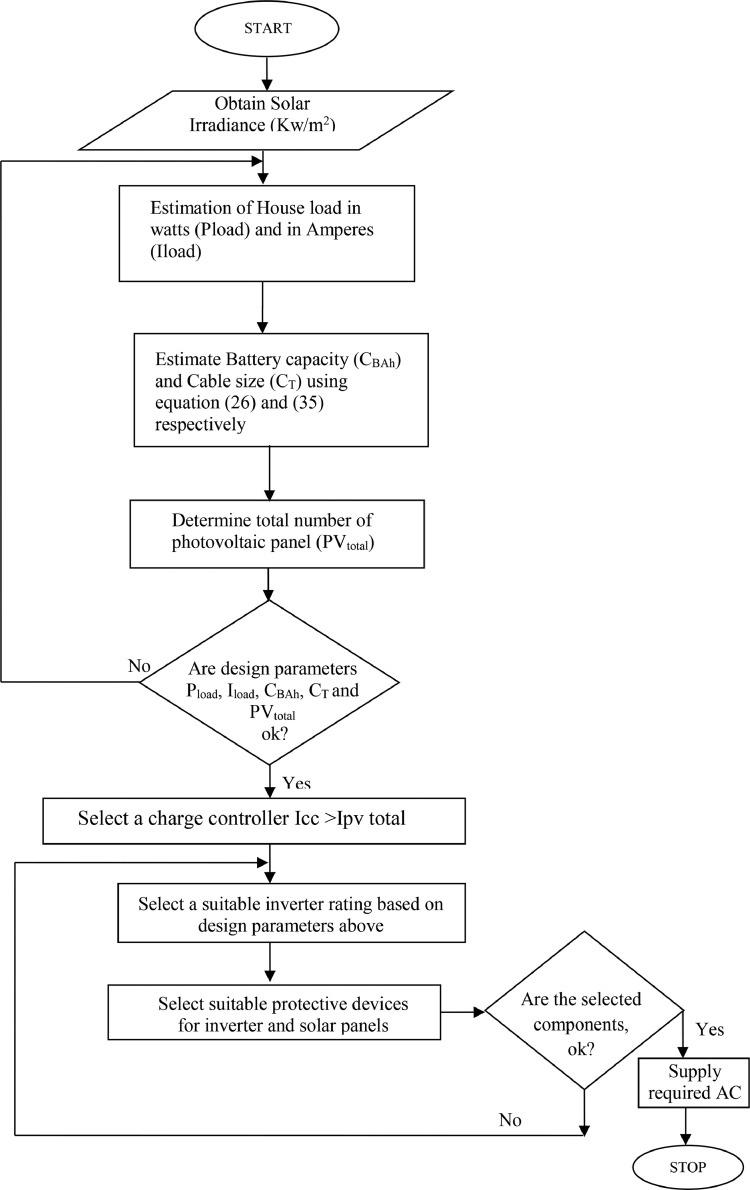
Design Flowchart.

Fig. 3 and 4 presents the block diagram and the designed/implementation flowchart showing the flow process of an off-grid photovoltaic solar system. Performance analysis was carried out on a 2 kW solar PV installation for a residential building. It involves the use of energy obtained from the internal monitoring of the charge controller. The load measured was subsequently used to select a suitable inverter that was used for the implementation of the proposed stand-alone solar PV system to be installed in the building. This study considered other sources of energy like utility power supply and petrol driven engine (i.e., generator) as alternative to solar power in the residential building used as case study.

### Block diagram representation

The details of the photovoltaic components used in this research as well as the block diagram's contents displayed in Fig. 3.

### Case study design and implementation approach

The entire research work was carried out using the following procedural steps:

a. Load estimation
b. Selection of inverter
c. Battery - bank determination
d. Design analysis of the solar panels
e. Choosing an appropriate Charge Controller
f. Choosing the Right cable and size
g. BEME (bills of engineering measurement and evaluation).
h. Testing and experimentation of the system.

In the absence of grid supply, off grid system is the best alternative. During the day, the PV power supplies the energy required and at night the energy stored in the batteries are being utilized. Fig. 4 presents the complete flowchart of the proposed system.

In this diagram (Fig. 3), the arrows show the flow of current, and also the description of some items contained in a block.

#### Estimation of load

The house load estimation was obtained by substituting the load schedule data shown on Table 1 in Eq. (1). The overall actual load and the total watt-hour per day estimated are 2, 293 W and 23, 820 watt – hours respectively.

**Table 1:** Load schedule.

S/N	Appliances	Appliance	Quantity Categories	Actual Load (W)	Operating Hours (Hours per day)	Watt-Hour Per day
1	LED Lights	Night App	80	5	13	5200
2	Cell Phone Charger	24 Hours	4	5	24	480
3	TV Set	14 Hours	1	22	4	3080
4	P/Machine (Borehole) (30Mins)	0.5 Hours	1	800	0.5	400
5	Fans	24 Hours	4	120	24	11,520
6	W/Machine	2 Hours	1	320	2	640
7	Fridge	10 Hours	1	250	10	2500

This implies that,

TotalActualLoad=2,292W

TotalWatt−Hourperday=23,820

Applying diversity factor and selecting most frequent load in use which totaled to 11,260 Wh. This implies that the remaining loads are not frequently used by the client which is presented in Table 2.

**Table 2:** System load specification.

Serial No.	Appliance Name	Load (Watt - Hour)
1	Fridge	2500Wh
2	2 Fans	5760Wh
3	60 Lamps	3,900Wh
4	Pump	400Wh

Therefore, Total Load (Watt-Hour) = 12, 560Wh

#### Selection of inverter

From Table 1, the actual loads in kW = 2.292Kw = 2.3 kW, i.e., the total load on PV system =2.3 kW

Therefore, the choice of inverter size will be 2.3/0.8 = 2.875KVA and for future expansion an inverter of 3.5KVA was selected

Totalloadnotalwaysinuse=12,560Wh

Overallloadestimated=23,820Wh

Designload=23,820−12,560

=11,260Wh

NB: Solar irradiance of case study town in Nigeria called ILORIN for the year 2016–2021 which were obtained from Nigeria Meteorological Agency, Abuja- Nigeria are provided in Table 3 [21]

**Table 3:** Solar irradiance (kW/m^2^).

YEAR	2021	2016	2017	2018	2019	2020
MONTH						
JAN	7.8	7.6	6.6	8.3	7.2	7.6
FEB	7.1	7.1	7.1	7.3	5.4	7.1
MAR	7.1	7.2	7.3	7.8	6.3	7.3
APR	7.3	7.1	7.0	7.5	6.9	7.2
MAY	7.1	7.0	6.1	6.4	6.8	6.9
JUN	6.2	6.2	5.5	6.0	6.3	6.1
JUL	4.8	4.7	4.5	4.8	5.3	4.7
AUG	3.6	3.5	3.3	3.6	3.9	3.6
SEP	4.6	4.4	4.2	4.2	4.0	4.4
OCT	6.3	6.3	6.0	4.1	6.1	6.0
NOV	7.9	7.9	7.8	8.0	7.9	7.9
DEC	8.6	8.4	8.3	8.5	8.1	8.6
AVE. AN.	78.2	77.6	73.8	76.5	66.1	77.5

NB: AVE. .AN = Average Annual value.

In obtaining the Peak sun hour (PSH), Eq. (25) can be used [2,5].

(25) $$\text{PSH}=\;\frac{A}{M_{N}\times Y_{N}}$$

Where,

A= Sum of average monthly solar irradiance for a period of 6 years (From Table 3) i.e., 78.2 + 77.6 +…

M_N_= Total number of months (here it is 12 months),

Y_N_= Total number of years (6 years)

Therefore, by parameters’ substitution in Eq. (25) imply that

$$\text{PSH}=6.36\text{kwh}/m^{2}$$

This simply mean that the solar irradiance of the case studied City in Nigeria (Ilorin) = 6.36kw/m^2^.

#### Battery - bank determination

Battery choice is considered using the following three parameters: depth of discharge, load - time and capacity. Batteries are manufactured such that it discharges during usage and made to be rechargeable after usage. They are rated in ampere hours (AH). In applications where more than one battery is required to be used, they are often wired in series, parallel or series - parallel to achieve the desired voltage level as well as the desired ampere hours. Therefore, selection of battery bank for daily energy generation per day was achieved using Eq. (26) [22]:

(26) $$C_{BAh}=\left(E_{db}\times DOA\right)/\left(DOD\times ..BA\times V_{B}\;\right)$$

Where,

E_db_= Daily energy required from battery = 11,260

C_Bah_= Battery Rating

DOA= Days of Autonomy =1

DOD= Depth of discharge = 80 percent

ƞ_BA_= Efficiency of Battery Amperes = 80 percent

V_B_= Chosen DC nominal Voltage of the block battery = 24

Therefore, by parameters’ substitution in Eq. (26) imply that $C_{\text{BAh}}=733\mathrm{AH}$

Consequently, 800AH battery was selected.

Also, the required series and parallel batteries connections can be obtained using Eq. (27) to (29) that follows [23]:

(27) $$\text{Number}\;\text{of}\;\text{Parallel}\;\text{Batteries}\;\left(N_{Pb}\right)=\frac{\text{Total}\;\text{Battery}\;\text{Rating}\;\left(\text{AH}\right)}{\text{Battery}\;\text{Rating}\;\left(\text{AH}\right)}=\frac{C_{\text{tb}}}{C_{b}}$$

(28) $$Number\;of\;series\;batteries\;\left(N_{Sb}\right)=\frac{VDC}{V_{b}}=\frac{System\;Voltage}{Battery\;Voltage}$$

(29) $$\text{Total}\;\text{Number}\;\text{of}\;\text{Batteries}\;\left(N_{tb}\right)=N_{Sb}\times N_{Pb}$$

Therefore, Eqs. (27), (28) and (29) give the battery details as follows.

**Total number of parallel batteries**

From Eq. (27),

$$N_{Pb}=\frac{400}{200}=2$$

Total number of parallel batteries used was 4.

A 200AH Block battery was selected.

**Total number of series batteries**

From Eq. (28),

$$N_{Sb}=\frac{24}{12}=2$$

**Total number of batteries**

From Eq. (29),

$$N_{tb}=2\;\times \;2=4$$

Therefore, total number of batteries used was 4 × 200AH batteries.

#### Design analysis of the solar panels

In calculating the Solar Panels Wattage, Eq. (30) was used [22].

(30) $$P_{\text{PV}}=\frac{E_{d}}{\text{PSH}\;\times \eta_{\text{CR}}\times \eta_{\text{inv}}}$$

Where,

E_d_= Energy consumption of Residential building per day = 11,260 Watt - hour per day

ƞ_CR_= Assumed charge controller efficiency = 95 percent

ƞ_inv_= Assumed Inverter efficiency = 95 percent

PSH= Average annual peak sunshine hours (PSH) for Ilorin between 2016 - 2021 gives 6.3kwh/m^2^ [21].

Therefore, by parameters’ substitution in Eq. (30) imply that $P_{\text{PV}}=1961W$

Consequently, the total panel capacity selected and used was 2000 W

(31) $$Total\;DC\;Current,\;I_{t}=\frac{Average\;Peak\;Power\;\left(Watts\right)}{System\;Voltage\;\left(V_{SP}\right)}$$

(32) $$Number\;of\;Series\;Module\;\left(Ns\right)=\frac{System\;Voltage\;\left(V_{SP}\right)}{Max.\;Panel\;Voltage\;\left(Panel\;Short\;Circuit\;Voltage\right)}$$

(33) $$Number\;of\;Paralled\;Module\;\left(Np\right)=\frac{I_{t}}{I_{m}}$$

Where I_m_ = Module Current or PV Current

(34) $$N_{t}=\;N_{S}\times \;N_{P}$$

Thus, in this research, Eq. (31) to (34) was used in determining the number of series and parallel panels connection. Therefore,

$$I_{t}=\frac{2000}{60.30}=33A$$

System voltage Vsp here is the panel design voltage which was 60.32 V (short-circuit voltage) and it has an open circuit voltage of 75V DC.

$$N_{S}=\frac{60.30}{30.15V}=2$$

$$N_{P}=\frac{33}{8.9}=4$$

$$N_{t}=\;2\;\times \;4=8$$

**Fig. 5:**
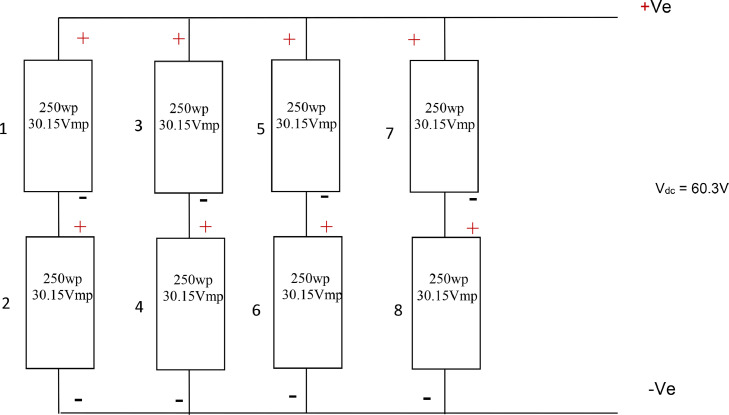
Schematic Diagram of Solar Panel Arrangement.

Therefore, total number of panels used was 8 Panels rated 250 W (8pieces). The arrangement and connections of these panels is shown in Fig. 5.

#### Choosing an appropriate charge controller

Obtain PV Array current I.e., the minimum charge controller Input current. This can be obtained using Eq. (35).

(35) $$\text{PV}\;\text{Array}\;\text{Current}\;=\;I_{\text{SC}\;}x\;P_{\text{VP}}\;x\;\text{SF}$$

Where,

I_SC_= Panel short circuit current = 8.9 A

P_VP_= Panels in Parallel = 4

SF= Safety Factor (SF is assumed equals 1.25)

Since Eq. (35) is similar to Eq. (23), therefore, substitute Isc=8.9AandE1 = 4 in using Eq. (23), then,

PV Array Current = 8.9 × 4 × 1.25 = 44.5A

PV Array Current = 60 A is the nearest available rating; Hence, a 24 V/60A charge controller was chosen.

#### Choosing the right cable and size

In this research work, cable with copper conductors was used to achieve high system reliability and effective performance of the system. Solar cables are of different sizes depending on the cross-sectional area of the conductors. Eq. (36) is often used to determine the size of the cable to be used [12]:

(36) $$\text{CT}=\frac{\left(L\;\times \;I\;\times \;0.04\right)}{\left(V_{\text{SA}}/20\right)}$$

Where,

CT= Size of cable

L= Desire length of cable

I= Array current

V_SA_= Solar Array Voltage

Solar array voltage is the voltage generated from the panels when connected in series parallel. However, in this research, V_SA_ was obtained to be 60.5 V. Furthermore, the following parameters were used in achieving the required cable size in Eq. (36), I.e., *L* = 20 m, *I* = 44.5A and V_SA_ = 60.5 V. Therefore, substituting these parameters in Eq. (36) gives value of $\text{CT}=12mm^{2}$. Consequently, the available cable size closer to 12mm^2^ is 16mm^2^; therefore, a 16mm^2^cable was selected.

### Pictures of the constructed work

**Fig. 6:**
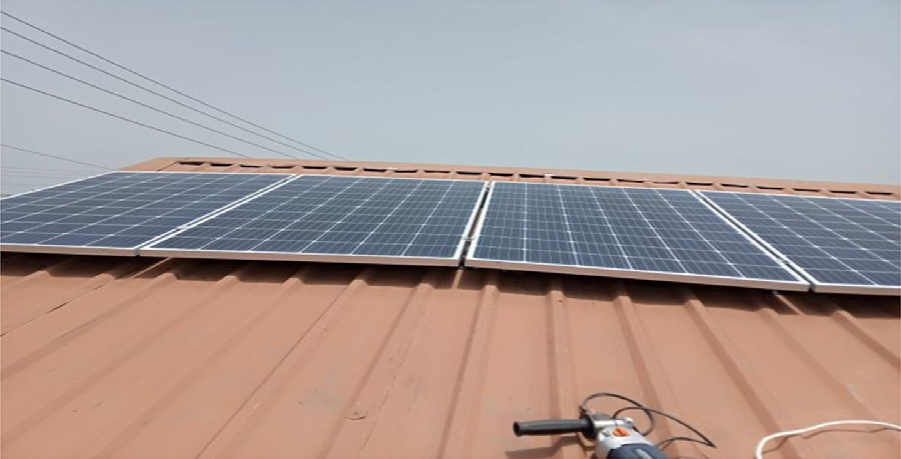
Solar PV array.

**Fig. 7:**
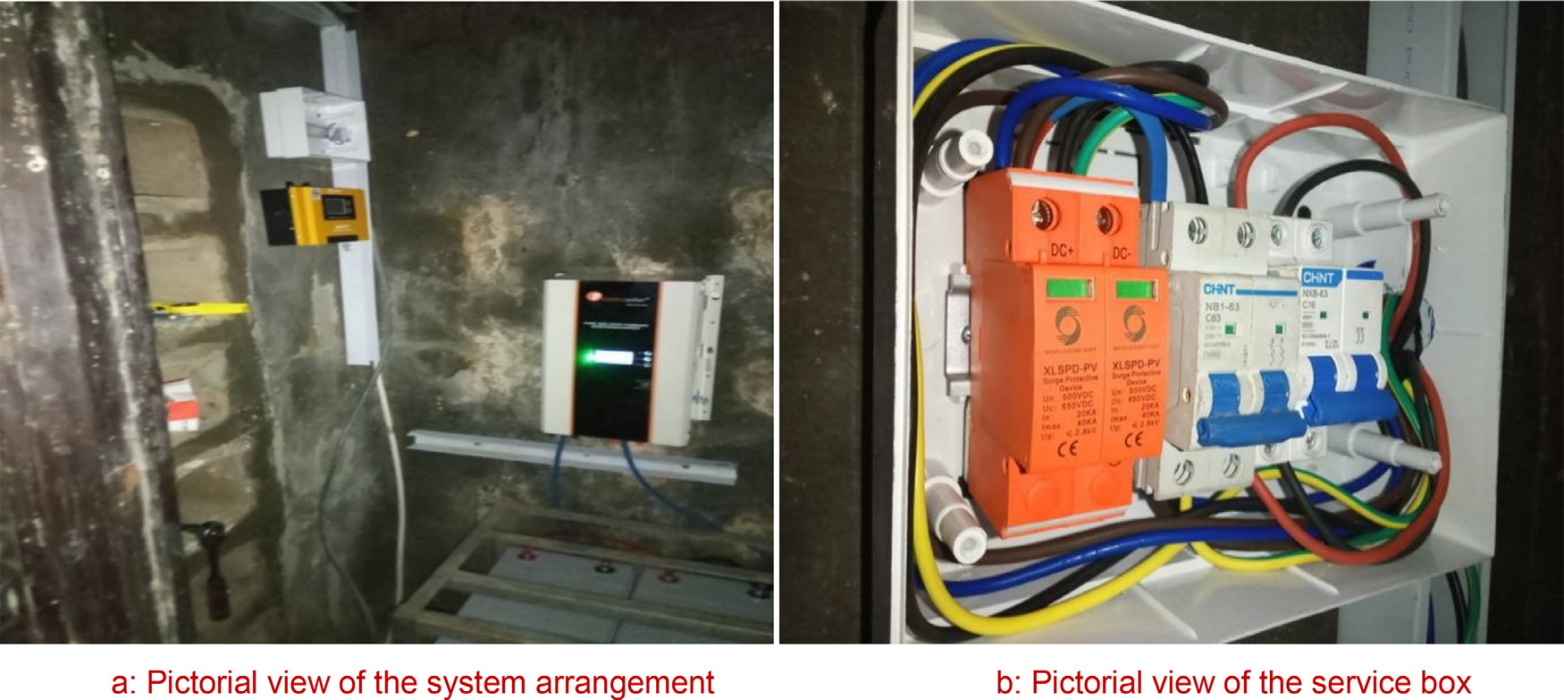
**a**: Pictorial view of the system arrangement; **b**: Pictorial view of the service box.

Prior to the commencement of the construction work, preparation carried out includes, getting the best position of installation using the compass, the tilt angle, measuring the position of best fit for the rayless mounting structures, cutting of flash bands to cover areas of possible leakages after installation as demonstrated in Fig. 6. This was followed with electrical wiring and protective devices mounting and installation as shown in Fig. 7.

### KVA installed stand-alone solar pv description

This system comprises of components that include estimated loads, inverter, solar panel, charge controller, battery, mounting structure, and the protective devices. The inverter used is an industrial grade inverter with pure sine wave output having battery charger and a built-in liquid crystal display (LCD) for easy monitoring. This is the type of inverter that incorporate mechanism that prevent fluctuations in the battery current and also serves as an automatic voltage regulator. The two series and four parallel PV panels are AEM specification each rated 250 W maximum output power taken at 1000 W/m^2^ solar irradiance. The PV materials and the displayed workmanship have a guarantee of 5 years.

#### Testing and results

**Fig. 8:**
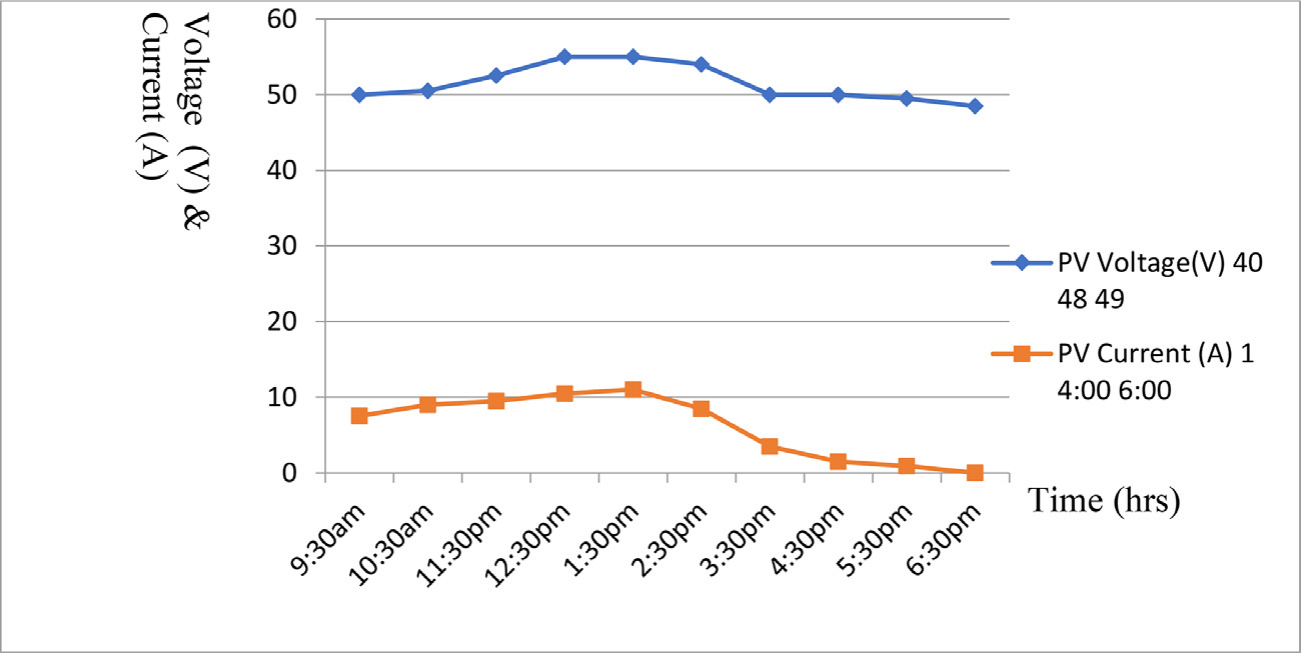
Solar Cell curves for both voltage and current against time between Aug. to Oct.

**Fig. 9:**
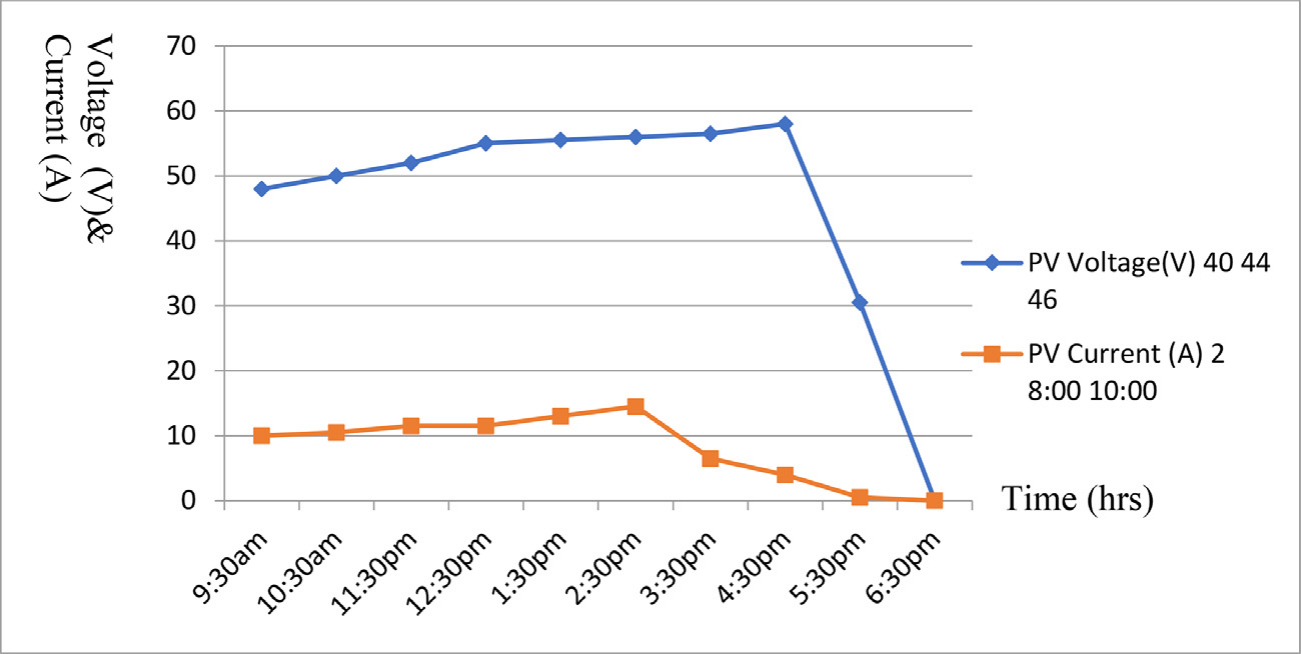
Solar Cell curves for both voltage and current against time between Nov. to Jan.

After mounting the completesolar installation, some technical results were recorded. Table 4 to 5 shows part of the results obtained while Fig. 8 to 9 shows the graphical illustration of the results obtained from the aforementioned tables. However, prior to mounting panels on the roof, average solar cell results for hourly current generated were carried out at a date in each quarter of the year i.e., August - October, November - January, February - April and May - July as shown in Table 4 to 7. Furthermore, some abbreviations were also used in Table 4 to 7 to describe the parameters involved. These abbreviations include: PVR, PVV, PVC, B, and L, and their interpretations are presented in Table 4 to 7 as follows; PVR - PV Rating, PVV - PV Voltage, PVC - PV Current, B- Battery, and l- Load.

**Table 4:** Average solar cell result obtained for hourly current generated.

Date	Time (Hours) (W)	PVR (V)	PVV (A)	PVC (V)	B (W)	L
AUG-OCT	6:30am	800	40.00	01.00	23.00	500
	7:30am		48.00	02:00	24.00	500
	8:30am		49.00	06:00	24.50	500
	9:30am		50.00	07.50	24.50	500
	10:30am		50.50	09.00	25.00	500
	11:30pm		52.50	09.50	26.00	500
	12:30pm		55.00	10.50	27.50	500
	1:30pm		55.00	11.00	27.50	500
	2:30pm		54.00	08.50	27.00	500
	3:30pm		50.01	03.50	26.00	500
	4:30pm		50.00	01.50	25.50	500
	5:30pm		49.50	00.90	25.00	500
	6:30pm		48.50	00.00	24.50	500

**Table 5:** Average solar cell result obtained for hourly current generated.

Date	Time PVR (Hours) (W)	PVV (V)		PVC (A)		B (V)		L (W)	
NOV-JAN	6:30am	540	40.00		02.00		23.00		500
	7:30am		44.00		08:00		24.00		500
	8:30am		46.00		10:00		24.50		500
	9:30am		48.00		10.00		24.50		500
	10:30am		50.00		10.50		24.50		500
	11:30pm		52.00		11.50		25.00		500
	12:30pm		55.00		11.50		25.50		500
	1:30pm		55.50		13.00		26.00		500
	2:30pm		56.00		14.50		26.20		500
	3:30pm		56.50		06.50		26.60		500
	4:30pm		58.00		04.00		27.00		500
	5:30pm		30.50		00.50		25.00		500
	6:30pm		00.00		00.00		24.50		500

Table 4 was obtained based on the following parameters i.e., SOC = 95% at 7am, DOD =5% at 7am, Sun hours/day = 9 hrs and Temperature 29 °C/31 °C. The table also showed the average sun hour's data obtained between the months of August to October 2021. Table 5 was obtained based on the following parameters i.e., SOC = 95% at 7am, DOD =5% at 7am, Sun hours/day = 9 hrs and Temperature 31 °C/36 °C.The table showed the average sun hour's data obtained between the months of November 2021 to January 2022. Table 6 was obtained based on the following parameters i.e., SOC = 88% at 7am,DOD =12% at 7am,Sun hours/day = 7 hrs and Temperature 26 °C/35 °C. The table showed the average sun hour's data obtained between the months of February to April 2022. Table 7 was obtained based on the following parameters i.e., SOC = 90% at 7am, DOD =10% at 7am, Sun hours/day = 10 hrs and Temperature 24 °C/33 °C. The table showed the average sun hour's data obtained between the months of May to July 2022.

**Table 6:** Average solar cell result obtained for hourly current generated.

Date	Time (Hours)	PVR (W)	PVV (V)	PVC (A)	B (V)	L (W)
FEB-APR	6:30am	1080	45.00	03.00	23.50	500
	7:30am		45.50	12:50	24.50	500
	8:30am		47.00	13:00	25.00	500
	9:30am		48.00	14.00	25.50	500
	10:30am		49.00	15.00	25.50	500
	11:30pm		51.00	16.00	26.00	500
	12:30pm		54.00	18.50	27.00	500
	1:30pm		54.50	20.00	27.50	500
	2:30pm		55.70	21.00	28.00	500
	3:30pm		56.50	11.00	28.60	500
	4:30pm		57.00	09.00	27.00	500
	5:30pm		32.00	02.00	25.00	500

**Table 7:** Average solar cell result obtained for hourly current generated.

Date	Time (Hours)	PVR (W)	PVV (V)	PVC (A)	B (V)	L (W)
MAY-JUL	6:30am	1080	45.00	02.00	23.00	500
	7:30am		50.00	11:50	24.00	500
	8:30am		52.00	12:00	25.00	500
	9:30am		55.00	13.00	26.90	500
	10:30am		57.00	14.00	27.10	500
	11:30pm		60.00	15.00	28.50	500
	12:30pm		37.00	17.50	27.50	500
	1:30pm		67.20	19.50	28.40	500
	2:30pm		66.90	10.00	28.30	500
	3:30pm		65.0	18.00	28.30	500
	4:30pm		50.00	07.00	27.30	500
	5:30pm		34.50	03.00	25.50	500
	6:30pm		00.00	00.00	25.00	500

**Fig. 10:**
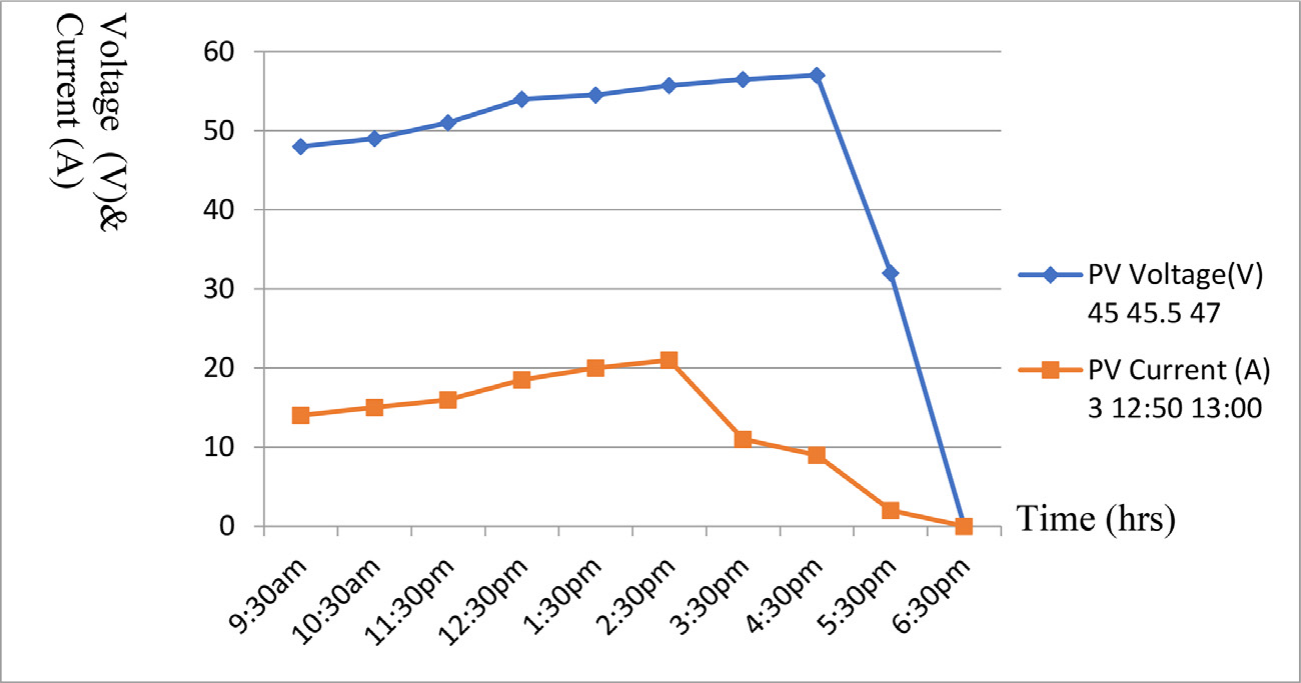
Solar Cell curves for both voltage and current against time between Feb. to April 2022.

**Fig. 11:**
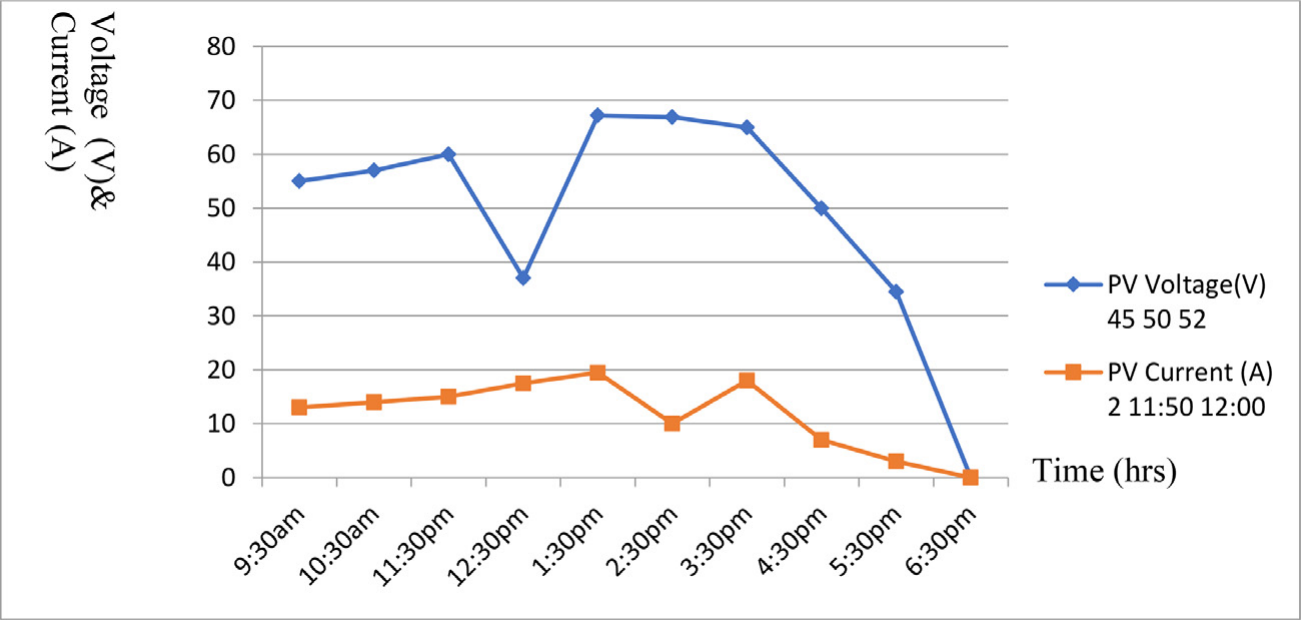
Solar Cell curves for both voltage and current against time between May to July 2022.

Fig. 8 illustrate a plot of Table 4 with voltage and current against time for August to October 2022. The graph shows the average sunshine hour between the months of August to October 2021. Fig. 9 illustrate a plot of Table 5 with voltage and current against time for November 2021 to January 2022. The graph shows the average sunshine hour between the months of November 2021 to January 2022. Fig. 10 illustrate a plot of Table 6 with voltage and current against time for February to April 2022. The graph shows the average sunshine hour between the months of February to April 2022. Fig. 11 illlustrates a plot of Table 7 with voltage and current against time for May to July 2022. The graph shows the average sunshine hour between the months of May to July 2022.

#### Method validation

Fig. 8 illustrate a plot of Table 4 with voltage and current against time for August to October 2022. The graph shows the average sun shine hour between the months of August to October 2021. Fig. 9 also illustrate a plot of Table 5 with voltage and current against time for November 2021 to January 2022.It further shows the average sun shine hour between the months of November 2021 to January 2022. Fig. 10 display a plot of Table 6 with voltage and current against time for February to April 2022. It also shows the average sunshine hour between the months of February to April 2022. Fig. 11 illustrate a plot of Table 7 with voltage and current against time for May to July 2022. It shows the average sunshine hour between the months of May to July 2022.

When the solar cell is not connected to any load, the recorded load current will be minimum or approximately zero, while the voltage across ii is maximum and referred to as open circuit voltage (Voc) as shown in Fig. 8 to Fig. 11. But when the solar cell is short circuited I.e., the positive and negative leads connected, the voltage across the cell (V_OC_) is approximately zero or at minimum but the current flowing out of the cell is large and often referred to as short circuit current (I_SC_). The PV panels were connected in series and parallel combinations to increase the voltage and current capacity of the solar array. It is important to note that when the solar panels were connected in only series combination, the voltage increased but when such solar panels were connected in parallel, the current increased. Furthermore, it was generally observed that when the system was loaded, the excess voltage was converted into current up to the tune of 40A, implying that voltage dropped from 75VDC to 30VDC and current appreciates from 10A to 40A respectively for a load of 1000 W upward.

## Conclusion

The off-grid solar PV system can provide an uninterrupted power for a residential building. All the power the system needs will be generated by a solar panel that will store its charge with block battery. The proposed 3.5 kVA Solar PV system was used to supply residential building in Ilorin, Kwara state, Nigeria, so as to avoid the possible changes that can prevent the effects of carbon from generators. Nigeria being a developing country where the power generation output is below 4000 MW which stands very much inadequate. With abundant availability of sun, Nigeria has the potential of generating electricity by solar power technology. With this, solar power can easily be generated throughout the year but best practiced when the sunshine is maximum. The paper has presented a diverse method of designing and implementation of a 3.5 kVA off-grid solar system for a typical Nigerian bungalow living apartment. A simple flowchart that accurately described the method is presented and discussed. The results obtained clearly shows that the solar PV system Installed was able to produce the energy required per day. The results verify the property of the proposed PV system design for solving the power supply in the selected residence in Ilorin. The block battery selected after the design was able to support the load for about 24hours with a specified load chosen in Table 2. This was made possible as the daily yield of the panels between January to December was between 1400wp to 1900wp per day because of the panel arrangement chosen. This design was able to provide AC supply with high quality backup throughout the day.

## Future work

It is suggested that a design on off-grid PV Solar system for a commercial building should be carried out; such that the system behavior over the months when the solar radiation is at their minimum and when it is at its maximum will be tested so that complete and extensive results will be obtained for a full year or seasonal when heavy loads are subjected to the system without any diversity factor. All these changes in loads application will result in more conclusive and pronounced outcomes for the future optimization of off-grid PV systems. A thorough cost for such heavy design should also be investigated so as to compare it cost with what is available in the market.

## Funding

Not applicable

## Data availability and materials

Data for Average Sunshine Hours between years 2016–2021 collected from the Nigerian Meteorological Agency (NIMET) has already been acknowledge.

## CRediT authorship contribution statement

The paperwork was handled by the following authors. Searching for appropriate data and the framework of design was taken care by LM. LM, OO, and KM worked together on the installation of the solar components at the chosen case study Venue. KM also coordinated results collation. Post field work was done by OO and LM prepared the first drafted manuscript aimed at submission to Journal. At the end, the three authors finally read the manuscript and agreed that the correction fashion of the paper be sent to our choice of Journal.

## Declaration of Competing Interest

The authors declare that they have no known competing financial interests or personal relationships that could have appeared to influence the work reported in this paper.
